# Measuring personality functioning with the 12-item version of the OPD-Structure Questionnaire (OPD-SQS): reliability, factor structure, validity, and measurement invariance in the general population

**DOI:** 10.3389/fpsyg.2023.1248992

**Published:** 2023-09-14

**Authors:** Johannes C. Ehrenthal, Johannes Kruse, Bjarne Schmalbach, Ulrike Dinger, Samuel Werner, Henning Schauenburg, Elmar Brähler, Hanna Kampling

**Affiliations:** ^1^Department of Psychology, University of Cologne, Cologne, Germany; ^2^Department of Psychosomatic Medicine and Psychotherapy, Justus Liebig University Giessen, Giessen, Germany; ^3^Department for Psychosomatic Medicine and Psychotherapy, Medical Center of the Philipps University Marburg, Marburg, Germany; ^4^Department of Medical Psychology and Medical Sociology, University Medical Center of the Johannes Gutenberg University Mainz, Mainz, Germany; ^5^Department of Psychosomatic Medicine and Psychotherapy, Medical Faculty of the Heinrich Heine University Düsseldorf, Düsseldorf, Germany; ^6^Department of General Internal Medicine and Psychosomatics, University Hospital Heidelberg, Heidelberg University, Heidelberg, Germany; ^7^Department of Psychosomatic Medicine and Psychotherapy, University Medical Center of the Johannes Gutenberg University, Mainz, Germany; ^8^Department of Medical Psychology and Medical Sociology, University Hospital Leipzig, Leipzig, Germany

**Keywords:** personality functioning, OPD-SQS, personality disorders, assessment, norm values, validity

## Abstract

**Background:**

The assessment of personality functioning is at the core of current dimensional models of personality disorders. A variety of measures from different clinical and research traditions aim to assess basic psychological capacities regarding the self and others. While some instruments have shown reliability and validity in clinical or other selected samples, much less is known about their performance in the general population.

**Methods:**

In three samples representative of the German adult population with a total of 7,256 participants, levels of personality functioning were measured with the short 12-item version of the Operationalized Psychodynamic Diagnosis – Structure Questionnaire (OPD-SQS). We addressed questions of factor structure, reliability, validity, factorial invariance, and provide norm values.

**Results:**

Confirmatory factor analysis indicated a satisfactory to good model fit. OPD-SQS models were mostly unaffected by variables such as gender, age, or measurement time. As expected, personality functioning was associated with general psychopathology as well as indices of occupational functioning.

**Conclusion:**

The OPD-SQS is a viable measure to assess personality functioning in the general population.

## 1. Introduction

Measuring severity of personality disorders (PD) through levels of personality functioning has attracted researchers and clinicians alike. From a clinical perspective, it enables treatment-planning on a personalized profile of abilities and personality styles rather than on categorical diagnosis (Morey and Hopwood, 2020). From a research-perspective, it solves several issues related to variance and distributions, cut-off values, comorbidity, and unspecific residual categories. However, there are a number of challenges associated with this approach as well, some of them on a conceptual level, others in relation to a lack of empirical data. While the number of studies in clinical populations is growing, less is known about the measurement of personality functioning in the general population.

The DSM-5 Alternative Model for the Assessment of Personality Disorders [AMPD; (Morey et al., 2011)] and the revision of the related chapter of the ICD-11 (Tyrer et al., 2011) have proposed a hybrid model for diagnosing PDs. Both approaches include a dimensional rating of basic psychological capacities as a stand-alone-measure as well as a prerequisite for further differentiation by clinical five-factor variables and PD prototypes (Tyrer et al., 2019). At the core of each model is a dimensional rating of severity of personality dysfunction, which has a twofold purpose: on the one hand, it serves as a general indicator of the level of capabilities in basic psychological functioning, and therefore helps to translate clinical phenomena into a measure of impairment, including cut-off scores for the presence or absence of a PD in the sense of categorical diagnosis. On the other hand, it includes clinically relevant information in itself, as DSM-5 and ICD-11 approaches define personality functioning as a combination of a number of capacities with regard to the self and interpersonal processes, which can inform treatment planning and evaluation. Since the initial publications, both DSM-5 AMPD as well as ICD-11 PD have stimulated a considerable amount of research (Zimmermann et al., 2019; Bach and Anderson, 2020; Krueger and Hobbs, 2020; Rek et al., 2020). In addition to measures specifically designed to capture dimensions of the DSM-5 and ICD-11, there are a number of approaches that assess similar constructs. One of those approaches is based on the Operationalized Psychodynamic Diagnosis system (OPD).

The OPD was initially developed by a group of researcher-clinicians from psychiatry, psychosomatic medicine, and clinical psychology in the beginning of the 1990s (Task Force, 2008; Arbeitskreis zur Operationalisierung Psychodynamischer Diagnostik, 2014). Its second edition (OPD-2) on which the questionnaire of the current study is based, is a multiaxial system to supplement psychiatric symptom-oriented diagnosis (OPD-2 Axis V) by adding treatment-relevant information in the areas of experience of illness and prerequisites for treatment (Axis I), interpersonal relations (Axis II), intrapsychic motivational conflicts (Axis III), and personality functioning, which has been labeled as ‘structure’ (Axis IV) in the OPD (Wöller and Kruse, 2018). The clinical model focuses on interrelations between axis II, axis III, and axis IV. Repetitive maladaptive interpersonal patterns are either based on conflictual insecure core motives which translate into ambivalent relationships that include motive-related wishes as well as a ‘defense’ against these wishes, or on impairments in personality functioning that complicate relationships on a basic level, or on a combination of both (Schauenburg and Grande, 2012; Ehrenthal and Benecke, 2019). “Structure” according to the OPD-2 operationalizes personality functioning by means of Levels of Structural Integration (LSIA).

The OPD-2 defines ‘structure’ as basic psychological capacities that are necessary for an adaptive organization of the self and its relation to internal and external ‘objects’, i.e., self and interpersonal functioning (Arbeitskreis zur Operationalisierung Psychodynamischer Diagnostik, 2014). The OPD-2 LSIA follows several principles: 1. It has a developmental focus and assesses functioning that is usually acquired in normal development but impaired due to disadvantageous conditions often related to neglect and abuse. 2. It assesses functioning with regard to abilities, not necessarily concerning specific behavioral consequences *per se*. In other words, it focuses on tools and skills, which result in a higher or lower probability for achieving adaptive behavior. 3. It tries to be as descriptive as possible, stepping back from wording directly associated with specific psychodynamic or other therapy schools. 4. Every facet is formulated as a possible direct treatment target that can be communicated with patients as shared goals.

‘Structure’ is observed in four different areas that relate to capacities of cognition/perception, regulation, communication, and attachment, each with regard to the self and others, resulting in eight broad structural functions. Every one of those consists of three subdimensions or facets: for example, ‘self-perception’ comprises capacities of self-reflection, affect differentiation, and identity, while ‘regulation of relationships’ is composed of capacities for protecting relationships, balancing interests, and anticipation [for a full description, see for example (Ehrenthal and Benecke, 2019)]. Each of these facets and dimensions is rated on a four-point scale from high integration (rating of 1), via medium (rating of 2) and low (rating of 3) levels of integration, to disintegration (rating of 4), resulting in an overall score of structural integration. These ratings can be further differentiated by intermediate steps. Usually, a score of 2.5 or 3 indicates a predominant structural impairment of personality functioning. Ratings are based on a semi-structured interview as conducted in routine clinical practice.

Interview-based ratings of the OPD LSIA show generally good inter-rater reliability, convergent validity, and construct validity (Zimmermann et al., 2012; Ehrenthal and Benecke, 2019). In 2012, Ehrenthal and colleagues (Ehrenthal et al., 2012) published a self-report questionnaire to complementarily assess personality functioning from the patients’ perspective (OPD-Structure Questionnaire; OPD-SQ). Its full version correlates significantly with expert ratings of the LSIA (*r* = 0.62), and explains variance in the prediction of the number of DSM-IV SCID-II personality disorder diagnoses incrementally to the expert ratings (Dinger et al., 2014). It is associated with attachment insecurity and neuroticism, and differentiates between individuals currently not in psychotherapy treatment, patients in outpatient treatment, and patients in inpatient psychotherapy, even if statistically controlled for influences of general symptom load (Ehrenthal et al., 2012). It is also related to burnout in students (Bugaj et al., 2016), PTSD symptom severity (Baie et al., 2020), or other self-report measures of personality functioning in adults (König et al., 2016) and adolescents (Bock et al., 2018). Patients with borderline personality disorder (BPD) and comorbid depression had higher scores in the OPD-SQ than patients with depression alone (Köhling et al., 2016). Higher scores of the OPD-SQ but not a categorical diagnosis of BPD were associated with active negative emotions in psychotherapy inpatients (Dinger et al., 2021). OPD-SQ scores but not depression predicted less success in blood glucose normalization in the treatment of patients with type 2 diabetes mellitus (Ehrenthal et al., 2019), and could be related to gastrointestinal complaints (Berens et al., 2021) and irritable bowel syndrome (Berens et al., 2021), profiles of eating disorders (Rohde et al., 2019), bipolar disorder (Wagner-Skacel et al., 2020), vaginismus and dyspareunia (Koops et al., 2021), and has been applied in different areas of psychotherapy research (Ehrenthal et al., 2020; Nikendei et al., 2020; Zeeck et al., 2020; Knefel et al., 2021; Kraus et al., 2021; Immel et al., 2022). In addition, the OPD-SQ is highly correlated with state and trait measures of emotional intelligence as well as self-reported levels of personality functioning according to the DSM-5 (Jauk and Ehrenthal, 2021), and it mediates the association between childhood trauma and adult depression (Dagnino et al., 2020). The OPD-SQ can also be used as an item pool for computer adaptive testing (Obbarius et al., 2021).

A 12-item short version (OPD-SQS) was developed by Ehrenthal and colleagues (Ehrenthal et al., 2015). It comprises three highly correlated subscales that address abilities around the topics of self-perception, shaping contact, and key relationship models. In the initial validation study, reported fit indices of the model were acceptable to good [TLI = 0.93; CFI = 0.95; RMSEA (90% CI) = 0.10 (0.09–0.11)]. Patients with higher scores in the OPD-SQS reported more previous psychotherapeutic and psychiatric treatment, independent of general symptom load (Ehrenthal et al., 2015). The OPD-SQS correlates with an interview-based assessment of DSM-5 LPFS [*r* = 0.78; (Zettl et al., 2019)], impaired reflective functioning (Zettl et al., 2020), and other convergent measures of severity of personality problems (Obbarius et al., 2019; Zimmermann et al., 2020). OPD-SQS scores partially mediate the association between adverse childhood experiences and health-related variables such as distress, experience of somatic symptoms, and body dysmorphic concerns (Krakau et al., 2021), and have been used in other areas of clinical psychology and health psychology (Ernst et al., 2022; Graetz et al., 2022; Vierl et al., 2022; Bröcker et al., 2023; Ernst et al., 2023; Zara et al., 2023).

While research on DSM-5 and ICD-11 personality functioning and related constructs such as the OPD has flourished in clinical samples as well as easy-to-access convenience samples, much less is known about the general population. In fact, Zimmermann and colleagues (Zimmermann et al., 2019) demanded that “… representative samples from the general population should be collected to establish normative values, which will greatly enhance the interpretation of test scores in single-case scenarios.” In accordance with this task, we present representative norm values of the German adult population of a common instrument to assess personality functioning: the OPD-SQS (Ehrenthal et al., 2015). In addition, we tested several models to replicate and expand previous findings on factorial validity and reliability. Furthermore, we addressed measurement invariance with regard to gender,^1^ measurement year, and age group. Finally, we aimed at replicating associations with general psychopathology as well as relationships with occupational functioning, the latter operationalized as unemployment frequency, personal income, and household income, and possible interaction effects of general psychopathology and personality functioning on occupational functioning.

## 2. Methods

### 2.1. Participants and procedure

The studies were not formally preregistered. The present representative study samples were collected in the years 2013, 2016, and 2019 by the demographic consulting company USUMA by order of the University of Leipzig. Sample size per study was determined by the usual procedure of the company. A total of *N* = 7,549 participants was collected using a multistage sampling method based on electoral districts, households, and persons in the household. Households were selected via random route procedure, and household members were selected using the Kish selection grid. This procedure aimed at obtaining a representative sample of the German population in terms of sex, age, and education. We confirmed this by comparing the distributions with data provided by the Federal Statistical Office of Germany (Statistisches Bundesamt. Bevölkerung, 2019). Descriptive statistics are reported in Table 1. Only participants with sufficient German language skills and an age of 18 or older were included. All participants were interviewed face-to-face by an USUMA employee, who assessed their language skills prior to the interview, which also served as a data validity check. Prior to participating, all participants were informed of the investigation’s general purpose and procedure and that data storage would be anonymized. In addition, they received a detailed data protection statement. The study included questionnaires assessing mental well-being of respondents. However, since no medical or psychological interventions were applied, there was no risk involved for participants. In accordance with German law, all participants provided verbal informed consent. Additionally, the studies followed the ICC/ESOMAR International Code of Marketing and Social Research Practice. After being informed of the general purpose of the survey, participants filled out the questionnaires mentioned below.

**Table 1:** Sociodemographic data of the samples.

	Total		2013		2016		2019	
	*n*	%	*n*	%	*n*	%	*n*	%
	7,256	100.0	2,449	100.0	2,434	100.0	2,373	100.0
Sex								
Male	3,397	46.8	1,146	46.8	1,140	46.8	1,111	46.8
Female	3,859	53.2	1,303	53.2	1,294	53.2	1,262	53.2
Age groups								
<30	1,330	18.3	443	18.1	455	18.7	432	18.2
30–39	1,079	14.9	326	13.3	386	15.9	367	15.5
40–49	1,228	16.9	444	18.1	392	16.1	392	16.5
50–59	1,412	19.5	454	18.5	480	19.7	478	20.1
60–69	1,171	16.1	381	15.6	386	15.9	404	17.0
>69	1,036	14.3	401	16.4	335	13.8	300	12.6
Family								
Married	3,177	43.8	1,083	44.2	1,061	43.6	1,033	43.5
Separated	182	2.5	62	2.5	52	2.1	68	2.9
Unmarried	2,164	29.8	695	28.4	757	31.1	712	30.0
Divorced	1,026	14.1	343	14.0	341	14.0	342	14.4
Widowed	687	9.5	266	10.9	214	8.8	207	8.7
Missing values	20	0.3	0	0.0	9	0.4	11	0.5
Work								
Fulltime	3,099	42.7	980	40.0	1,047	43.0	1,072	45.2
Part time	977	13.5	304	12.4	333	13.7	340	14.3
Unemployed	670	9.2	262	10.7	229	9.4	179	7.5
Retired	1902	26.2	713	29.1	613	25.2	576	24.3
In training	587	8.1	190	7.8	193	7.9	204	8.6
Missing values	21	0.3	0	0.0	19	0.8	2	0.1
Education								
<10 years	2,401	33.1	914	37.3	787	32.3	700	29.5
10 years	2,817	38.8	916	37.4	939	38.6	962	40.5
>11 years	1800	24.8	532	21.7	630	25.9	638	26.9
Current student	218	3.0	77	3.1	72	3.0	69	2.9
Missing values	20	0.3	10	0.4	6	0.2	4	0.2

In the current study, there were some missing data for the OPD-SQS. Due to the large samples as well as prerequisites of some of the conducted analyses, we only included individuals with complete data regarding the OPD-SQS. This resulted in an exclusion of 293 individuals,^2^ leaving 7,256 cases to be analyzed.

### 2.2. Instruments

#### 2.2.1. Personality functioning

The OPD-SQS (Ehrenthal et al., 2015) is a 12-item version of the OPD-Structure Questionnaire (Ehrenthal et al., 2012) described above. Individuals respond to each question on a scale from 0 (“fully disagree”) to 4 (“fully agree”). Different from the full version, where mean values are computed, the OPD-SQS uses a sum score for an easier use in clinical settings, resulting in a range of possible values from 0 to 48. Higher numbers indicate more impairment in personality functioning. In addition, three subscales (self-perception, shaping contact, and relationship model) with four items each can be computed. Findings on reliability and validity are cited above.

#### 2.2.2. General psychopathology

The PHQ-4 (Löwe et al., 2010) is a very brief measure of depression and anxiety derived from the depression and anxiety scales of the Patient Health Questionnaire (Kroenke et al., 2009), which is often used to assess general levels of common psychopathology. Two items on depression and two items on anxiety are scored on a scale from 0 (“not at all”) to 3 (“nearly every day”), resulting in a range for the sum score from 0 to 12. Higher numbers indicate more depressive and anxiety symptoms. The PHQ-4 is widely used as a screening measure or in representative samples as an assessment of common psychopathology. In addition, it has been associated with symptoms related to impaired personality functioning such as self-harm (Müller et al., 2016) or adverse childhood experiences (Schilling et al., 2016). In our study, ω for the PHQ-4 was 0.874 across all samples.

#### 2.2.3. Occupational functioning

A predominantly occupational aspect of psychosocial functioning was assessed by reported unemployment frequency (“How often have you been unemployed including current unemployment?”), personal income (13 groups in total, ascending in predefined steps from no personal income to 5,000 Euro and more), and household income (following the same ascending logic from under 500 Euro to 5,000 Euro and more). Both income variables were assessed as they seem to be related to psychosocial functioning in personality disorders in the long term (Skodol, 2018).

### 2.3. Statistical analyses

All analyses were conducted in R, using the packages *lavaan* and *semTools* (Rosseel, 2012; Jorgensen et al., 2020). First, we conducted confirmatory factor analyses for all three subsamples separately and jointly. Since the response data exhibits a strong right-skew, we used robust diagonally weighted least squares estimation and theta parameterization [WLSMV in *lavaan* (Li, 2016)]. We then judged the closeness of model fit based on the common recommendations (Hu and Bentler, 1999; Schermelleh-Engel et al., 2003): χ ^2^ should be non-significant, *CFI* and *TLI* should be greater than 0.950, and *RMSEA* and *SRMR* should be smaller than 0.080. We investigated reliability using McDonald’s ω (McDonald, 1999). Because of the ordered categorical data assumption, we used Green and Yang’s formula to estimate the coefficient (Green and Yang, 2009). Next, we tested for measurement invariance to ensure the comparability of observed groups between sexes, age groups, and measurement points (Meredith, 1993). Some modifications to the typical procedure have to be made to account for the ordinal data structure (Millsap and Yun-Tein, 2004; Wu and Estabrook, 2016). That is, intercepts are fixed to zero in all groups for identification purposes, and one instead checks the invariance of thresholds across groups. The successive constraint levels, thus, refer to item thresholds, factor loadings, and residual variances. Chen (2007) provides cut-off values for these nested model comparisons: Δχ ^2^ should be non-significant, Δ*CFI* should be ≤ 0.010, and Δ*RMSEA* should be ≤ 0.015. We conducted ordered logistic regression analyses for associations between OPD-SQS, PHQ-4, and their interaction on the one, and unemployment frequency, personal income, and household income using the R package MASS (Venables and Ripley, 2010). We report Nagelkerke’s R^2^ as a measure of effect size for the related models. We report how we determined our sample size, all data exclusions (if any), and all manipulations. As the USUMA studies usually comprise a variety of instruments per measurement point, we can only report the measures of interest for this study.

## 3. Results

### 3.1. Item descriptive analyses

As can be seen from the descriptive item parameters reported in Table 2, all of the OPD-SQS’s items have positive skewness. This means that the majority of participants responded with low values (such as 0 or 1), and a smaller number of participants responded with options in the middle or the upper extreme of the scale.

**Table 2:** Descriptive statistics for OPD-SQS items and scales.

	Items	*Md*	*M*	*SD*	Skewness	Kurtosis	λ 2013	ω 2013	λ 2016	ω 2016	λ 2019	ω 2019
OPD-SQS Total		11	11.69	8.35	0.70	3.21		0.92		0.92		0.93
OPD-SQS self		1	2.09	2.86	1.59	5.22		0.88		0.86		0.89
OPD01	*I sometimes feel like a stranger to myself.*	0	0.41	0.76	1.99	6.72	0.87		0.85		0.88	
OPD02	*If I think too much about myself, I tend to get confused.*	0	0.51	0.85	1.68	5.24	0.86		0.85		0.87	
OPD05	*There is often such a chaos of feelings inside me that I could not even describe it.*	0	0.60	0.91	1.54	4.74	0.89		0.88		0.89	
OPD08	*Sometimes my feelings are so intense that I get scared.*	0	0.57	0.87	1.54	4.89	0.81		0.82		0.84	
OPD-SQS contact		3	3.75	3.17	0.75	3.05		0.81		0.80		0.80
OPD04	*I find it difficult to make others understand me.*	1	0.82	0.96	1.00	3.24	0.80		0.80		0.85	
OPD06	*I sometimes misjudge how my behavior affects others.*	1	1.12	1.04	0.56	2.45	0.76		0.77		0.82	
OPD10	*I find it hard to get in contact with other people.*	1	0.81	0.97	1.02	3.28	0.75		0.75		0.67	
OPD11	*I do not have good self-esteem.*	1	1.00	1.13	0.90	2.85	0.69		0.69		0.67	
OPD-SQS relationship model		6	5.85	3.72	0.26	2.46		0.82		0.83		0.82
OPD03	*It can be dangerous to let others get too close to you.*	1	1.22	1.12	0.52	2.39	0.72		0.77		0.79	
OPD07	*If others know a lot about me I often feel somehow controlled or observed.*	1	1.16	1.11	0.59	2.42	0.80		0.78		0.84	
OPD09	*I’ve been hurt badly because I misjudged someone.*	2	1.63	1.30	0.25	1.95	0.72		0.77		0.75	
OPD12	*My experience is: if you trust people too much you can get nasty surprises.*	2	1.84	1.15	0.06	2.36	0.75		0.77		0.67	

OPD-SQS, short form of the Operationalized Psychodynamic Diagnosis Structure Questionnaire; *Md*, median; *M*, mean; *SD*, standard Deviation; *λ*, standardized factor loading in confirmatory factor analysis; *ω* = reliability coefficient omega.

### 3.2. Confirmatory factor analysis

Using the procedure described in the method section, we then tested the OPD-SQS’s factorial validity. As can be seen in Table 3, model fit was acceptable in all three samples. The χ ^2^ test was significant in all cases, and the descriptive measures of fit were in an acceptable range. *CFI* never fell below 0.950, and *TLI* was only marginally below the threshold in one sample. *SRMR* evinced good fit with values around 0.050. *RMSEA* fell slightly above the 0.080 cut-off value in two samples and clearly above it in the third sample. Overall, fit measures indicated acceptable fit. Standardized factor loadings are reported in Table 2 for all three samples. Most loadings exceeded 0.70, indicating strong discrimination at the trait level. Across the board, ω had very good values considering the brevity of the three scales: not a single ω coefficient was below 0.800.

**Table 3:** Confirmatory factor analysis of the OPD-SQS.

Sample	*χ* ^2^	*df*	*p*	CFI	TLI	RMSEA	SRMR
2013	992.847	51	<0.001	0.968	0.958	0.087	0.043
2016	938.162	51	<0.001	0.969	0.960	0.085	0.047
2019	1549.954	51	<0.001	0.955	0.941	0.111	0.053

OPD-SQS, short form of the Operationalized Psychodynamic Diagnosis Structure Questionnaire.

To justify the construction of a total scale score, we also tested a bi-factor model (Reise et al., 2007; Chen et al., 2012) for the OPD-SQS across the combined sample. This model showed a further improved fit, χ ^2^ (Obbarius et al., 2019) = 1370.865, *p* < 0.001, *CFI* = 0.985, *TLI* = 0.977, *RMSEA* = 0.066, *SRMR* = 0.030. The ω _hierarchical_ coefficient of 0.834 indicated that the majority of the OPD-SQS’s variance can be traced to a general factor.

In addition, we computed a second-order factor model in which the subscale factors of the OPD-SQS load on a higher order general factor. This model fit was slightly worse than the bi-factor model, but was still acceptable with regard to three out of the four employed descriptive fit indices, χ ^2^ (Löwe et al., 2010) = 3214.042, *p* < 0.001, *CFI* = 0.965, *TLI* = 0.955, *RMSEA* = 0.092, *SRMR* = 0.046. Reliability of the second order construct was ω = 0.870 at the first level, and 0.925 at the second level. The former of the two coefficients represents the proportion of total variance explained by the general factor in the model-implied covariance matrix.

### 3.3. Measurement invariance and group-specific differences of the OPD-SQS

We then tested the OPD-SQS’ three-factor model for measurement invariance across measurement years, age groups, and participant sex.^3^ As can be seen in Table 4, Δ*CFI* and Δ*RMSEA* never exceeded their respective cut-offs. Thus, we accept the measurement process for the OPD-SQS’s model to be invariant across the three aforementioned grouping variables.

**Table 4:** Test of measurement invariance of the OPD-SQS.

	*χ* ^2^	*df*	Δ*χ*^2^	Δ*df*	*p*	CFI	ΔCFI	RMSEA	ΔRMSEA
Time
Configural invariance	3447.714	153				0.964		0.094	
Threshold invariance	3665.362	201	217.648	48	<0.001	0.962	−0.002	0.084	−0.010
Threshold + loading invariance	3568.768	219	−96.593	18	<0.001	0.963	0.001	0.080	−0.005
Threshold + Loading + residual invariance	3367.169	243	−201.599	24	<0.001	0.966	0.002	0.073	−0.007
Sex									
Configural invariance	3290.396	102				0.965		0.093	
Threshold invariance	3486.338	126	195.942	24	<0.001	0.963	−0.002	0.086	−0.007
Threshold + loading invariance	3276.050	135	−210.289	9	<0.001	0.966	0.002	0.080	−0.006
Threshold + loading + residual invariance	2943.815	147	−332.235	12	<0.001	0.969	0.004	0.072	−0.008
Age groups									
Configural invariance	3376.990	306				0.967		0.091	
Threshold invariance	3560.937	426	183.947	120	<0.001	0.966	−0.001	0.078	−0.013
Threshold + loading invariance	3213.243	471	−347.694	45	<0.001	0.970	0.004	0.069	−0.009
Threshold + loading + residual invariance	2830.033	531	−383.209	60	<0.001	0.975	0.005	0.060	−0.010

OPD-SQS, short form of the Operationalized Psychodynamic Diagnosis Structure Questionnaire.

Given invariance, we then checked for differences in observed OPD-SQS sum scores across the three grouping variables (see Table 5). Eight out of nine *F* tests were significant, but this is not surprising given the very large sample size. As can be seen with the proportions of systematic variance *η*^2^, only participant sex explained more than 1%, and only for the ‘self’ subscale. Overall, the OPD-SQS scores were thus largely unaffected by participant sex, age, and measurement time.

**Table 5:** ANOVA table of the OPD-SQS subscales.

	*F*	*df1*	*df2*	*p*	*η* ^2^
Total score					
Time	42.844	2	7,253	<0.001	0.012
Sex	69.602	1	7,254	<0.001	0.010
Age groups	1.756	5	7,250	0.118	0.001
Self					
Time	30.951	2	7,253	<0.001	0.008
Sex	80.784	1	7,254	<0.001	0.011
Age groups	7.927	5	7,250	<0.001	0.005
Contact					
Time	42.346	2	7,253	<0.001	0.012
Sex	29.827	1	7,254	<0.001	0.004
Age groups	2.632	5	7,250	0.022	0.002
Relationship model					
Time	26.284	2	7,253	<0.001	0.007
Sex	51.41	1	7,254	<0.001	0.007
Age groups	0.381	5	7,250	0.862	0

OPD-SQS, short form of the Operationalized Psychodynamic Diagnosis Structure Questionnaire.

### 3.4. Convergent validity

We report latent and observed correlations among the OPD-SQS subscales and between the OPD-SQS and the PHQ-4 (see Table 6). Latent and observed correlations between the subscales were very high (*r* ≥ 0.677 and 0.503, respectively). Considering the PHQ-4 as a related measure of psychopathology, all subscales showed the expected pattern of moderate to strong associations.

**Table 6:** Correlation matrix of the OPD-SQS and the PHQ-4.

	OPD-SQS self	OPD-SQS contact	OPD-SQS relationship model	OPD-SQS G*	PHQ-4
OPD-SQS self	1	0.677	0.503	0.822	0.552
OPD-SQS contact	0.868	1	0.629	0.891	0.482
OPD-SQS relationship model	0.681	0.820	1	0.856	0.376
OPD-SQS G*	0.893	0.984	0.796	1	0.539
PHQ-4	0.581	0.535	0.434	0.584	1

Observed correlations are above the diagonal, latent correlations are below the diagonal. *Latent correlations regarding the G factor stem from the second-order factor model. OPD-SQS, short form of the Operationalized Psychodynamic Diagnosis Structure Questionnaire. PHQ-4, four item version of the Patient Health Questionnaire.

### 3.5. Norm values

Finally, we report Table 7 with normative percentile values for the OPD-SQS’s subscales and the total score. Since sociodemographic variables and time of measurement had only negligible impact on the sum scores, we did not stratify the norm values in any way.

**Table 7:** Normative percentile values of the OPD-SQS general scale and subscales.

Sum Score	Normative percentile values OPD-SQS Total	Normative percentile values OPD-SQS “self”	Normative percentile values OPD-SQS “contact”	Normative percentile values OPD-SQS “relationship model”
0	8	46	19	10
1	10	58	30	14
2	14	68	42	22
3	18	76	53	29
4	22	82	63	38
5	26	87	72	47
6	31	90	80	57
7	36	93	86	66
8	41	96	91	76
9	45	97	95	83
10	50	98	97	88
11	54	99	98	93
12	59	99	99	96
13	62	100	100	98
14	66	100	100	99
15	70	100	100	99
16	74	100	100	100
17	77			
18	79			
19	82			
20	84			
21	86			
22	89			
23	90			
24	92			
25	94			
26	95			
27	95			
28	96			
29	97			
30	98			
31	98			
32	98			
33	99			
34	99			
35	99			
36	99			
37	100			

OPD-SQS, short form of the Operationalized Psychodynamic Diagnosis Structure Questionnaire.

### 3.6. Occupational functioning

Number of unemployment times was not assessed in the 2016 survey. In all available samples, PHQ-4 as well as OPD-SQS, but not their interaction, were significantly associated with a higher probability for a higher unemployment frequency, and with a lower probability for lower personal as well as household income, with generally small effects (see Table 8).

**Table 8:** Associations between personality functioning, common psychopathology, and occupational functioning.

		*β*	*SE*	*t*	*p*	*Nagelkerke’s R* ^2^
Unemployment frequency	PHQ-4	0.218	0.035	6.21	< 0.001	0.034
	OPD-SQS	0.204	0.032	6.38	< 0.001	
	Interaction	−0.016	0.023	−0.70	0.487	
Personal income	PHQ-4	−0.142	0.026	−5.39	< 0.001	0.036
	OPD-SQS	−0.245	0.024	−10.17	< 0.001	
	Interaction	−0.009	0.017	0.54	0.589	
Household income	PHQ-4	−0.223	0.027	−8.15	<0.001	0.048
	OPD-SQS	−0.218	0.024	−8.98	<0.001	
	Interaction	−0.020	0.017	−1.15	0.249	

OPD-SQS, short form of the Operationalized Psychodynamic Diagnosis Structure Questionnaire; PHQ-4, four item version of the Patient Health Questionnaire; Interaction, PHQ4 * OPD-SQS.

## 4. Discussion

In three samples representative of the German adult population with more than 7,000 participants, we found a 12-item self-report measure of personality functioning to be mostly independent of gender, age and assessment year. OPD-SQS scores were associated with general common psychopathology as well as indices of occupational functioning.

The results of reliability and confirmatory factor analyses closely resemble results from previously published studies. Internal consistency was high in the original evaluation in a mixed as well as a clinical sample [Cronbach’s *α* = 0.87–0.89; (Ehrenthal et al., 2015)], a Danish college counseling sample [*α* = 0.85; (Østergård et al., 2019)], a mixed clinical and control sample [*α* = 0.92; (Zettl et al., 2020)], and a clinical sample [*α* = 0.88–0.89; (Obbarius et al., 2019)]. Across studies and samples, the OPD-SQS seems to be of robust acceptable to high internal consistency. The same applies for the CFA, which is also within the range of the original evaluation (Ehrenthal et al., 2015) and the data from Obbarius and colleagues (Obbarius et al., 2019). Similar to Obbarius et al., fit indices improved when testing a bifactorial model. Taken together, our results add to the stability of a general factor resulting from three interrelated facets. We also found that this was largely unaffected by variables such as age, gender, or the year of our measurement points. Therefore, the scores can be reasonably compared for inferences with relation to the theorized constructs.

Correlations with general common psychopathology were high. To our knowledge, there is no published study that did not find similar associations. This confirms OPD-SQS personality functioning as a substantial statistical predictor of psychological distress. Previous studies found the OPD-SQ and OPD-SQS to be associated to other variables such as attachment, personality, and mentalization even when statistically controlling for the effect of general psychopathology (Ehrenthal et al., 2012, 2015; Zettl et al., 2020). However, we cannot conclude the direction of the association from the current dataset, and further research with longitudinal designs and intensive measurement is needed to disentangle the shared variance.

Associations with indices of occupational function (i.e., unemployment frequency, personal, and household income) were significant but small. This points toward the importance of personality functioning as a risk factor that limits an individual’s options to live up to their full potential. We found no significant interactions between OPD-SQS and PHQ-4, tentatively suggesting several pathways from what used to be called Axis I and Axis II in the DSM-IV on related variables. Associations between occupational functioning and personality functioning are complex and call for more sophisticated designs and measures that take into account objective as well as subjective measures, and also other areas that go beyond mere occupational functioning, including relational and interpersonal functioning (Frankenburg and Zanarini, 2011; Skodol, 2018; Cruitt and Oltmanns, 2019; Buer Christensen et al., 2020). In addition, other areas such as educational achievement could be of interest for future research. This would need to include other variable such as intelligence. While one study (Bock et al., 2018) did not find a relationship between intelligence and the long version of the OPD-SQ (*r* = 0.02) in a clinical sample of 147 adolescents, this has yet to be replicated in adult samples.

Strengths of this study are the use of a rigorous sampling strategy and the high quality of the data. To our knowledge, this is also the first study using three samples each representative of the respective adult population to address the questions of reliability and validity of the construct of levels of personality functioning. The results are robust and comparable to earlier studies from mixed and clinical samples. In addition, we computed norm values that can be used for comparison and help to classify individuals according to the similarity or deviation of their personal scores on the instrument. Limitations are a cross-sectional design that limits inferences about causality. Even more so, using longitudinal designs that take into account significant personal events would be more suited to test the impact of personality functioning as a vulnerability factor. One might also argue from a methodological perspective that it would be sufficient to test invariance for a general factor and not the three subscales, especially, as the dataset does not allow for more nuanced testing of associations in terms of external validity. However, we wanted to stick with the originally proposed factor model, and would again stress the need for more longitudinal studies. And last, but not least: by using samples representative of the German adult population, we do include individuals with higher as well as lower levels of personality functioning, of which some may even be in psychotherapeutic or psychiatric treatment. Therefore, especially when using it as a reference sample, it is important to keep in mind that it is not a purely non-clinical dataset, but rather one that represents current distributions of psychopathology as one would expect in the general population.

From a clinical perspective, it is important to note that the development of measures from different traditions does not need to be a disadvantage. Quite the contrary, as personality functioning seems to be captured adequately and largely comparably by a variety of questionnaires and interviews (Wright et al., 2022), it provides clinicians enough freedom to use the construct within their respective intervention models, which is important for acceptance and dissemination. In addition, first attempts to make these measures comparable have been published as well (Zimmermann et al., 2020; Obbarius et al., 2021). And last, but not least, the third edition of the Operationalized Psychodynamic Diagnosis system (OPD-3) has just been published (Arbeitskreis zur Operationalisierung Psychodynamischer Diagnostik, 2023). While some subscales of the LSIA have been altered, most of the core aspects of OPD-2 personality functioning remained in the manual. Due to this continuity as well as the assumption of personality functioning to be a core latent construct which can manifest in different facets, we consider the OPD-SQ and OPD-SQS still similarly valid with regard to the expert rating. Taken together, the OPD-SQS can be considered a robust brief measure to assess personality functioning in the general population.

## Data availability statement

Due to the nature of the data collection, the datasets are not published in an open science repository. The datasets with the codes for the statistical analyses can be made available from the first author, upon reasonable request.

## Ethics statement

The studies involving humans were approved by the Institutional Review Board of the Medical Faculty of Leipzig University, Leipzig, Germany (050/13‐11032013; 297/16ek; Az 145/19-ek). The studies were conducted in accordance with the local legislation and institutional requirements. The participants provided their written informed consent to participate in this study.

## Author contributions

JE: conceptualization, writing of the original draft, methodology, and data curation. JK: conceptualization and data curation. BS: methodology and statistical analyses. UD, SW, HS, and HK: conceptualization. EB: conceived and designed original surveys. All authors contributed to manuscript revision, read, and approved the submitted version.

## Conflict of interest

JE, UD, and HS are authors of the original OPD-SQ and OPD-SQS. The questionnaires are free of charge and these authors declare no commercial or financial relationship that could be construed as a potential conflict of interest.The remaining authors declare that the research was conducted in the absence of any commercial or financial relationships that could be construed as a potential conflict of interest.

## Publisher’s note

All claims expressed in this article are solely those of the authors and do not necessarily represent those of their affiliated organizations, or those of the publisher, the editors and the reviewers. Any product that may be evaluated in this article, or claim that may be made by its manufacturer, is not guaranteed or endorsed by the publisher.

## Footnotes

^1^As the assessment procedure did not take into account various aspects of gender, we can only conduct analyses to a binary male–female version of gender.

^2^To be more precise, of 90,588 (12 items x 7,549 participants) possible values of the OPD-SQS, less than 1% (658) were missing, which led to the abovementioned exclusion of less than 1% of the initial participants.

^3^As mentioned above, we can only conduct analysis with regard to a binary (male–female) variant of gender, which we use the word ‘sex’ for on a level of operationalization to describe a binary self-attribution by the participants.
